# In Vivo PK-PD and Drug–Drug Interaction Study of Dorzagliatin for the Management of PI3Kα Inhibitor-Induced Hyperglycemia

**DOI:** 10.3390/ph18060927

**Published:** 2025-06-19

**Authors:** Guanqin Jin, Kewei Zheng, Shihuang Liu, Huan Yi, Wei Wei, Congjian Xu, Xiaoqiang Xiang, Yu Kang

**Affiliations:** 1 Clinical Research Center, Obstetrics & Gynecology Hospital of Fudan University, Shanghai 200433, China; jinguanqin7889@fckyy.org.cn; 2 Shanghai Key Lab of Female Reproductive Endocrine Related Diseases, Shanghai 200433, China; 3 Shanghai Key Lab of Reproduction and Development, Shanghai 200433, China; 4 Department of Gynecologic Oncology, Fujian Maternity and Child Health Hospital, College of Clinical Medicine for Obstetrics & Gynecology and Pediatrics, Fujian Medical University, Fuzhou 350001, China; 5 Fujian Province Key Clinical Specialty for Gynecology, Fujian Key Laboratory of Women and Children’s Critical Diseases Research, National Key Gynecology Clinical Specialty Construction Institution of China, Fuzhou 350001, China; 6 Department of Translational Medicine, Shanghai Jiatan Pharmatech Co., Ltd., Shanghai 201203, China; 7 Department of Obstetrics and Gynecology, Shanghai Medical School, Fudan University, Shanghai 200032, China; 8 Department of Clinical Pharmacy and Pharmacy Administration, School of Pharmacy, Fudan University, Shanghai 201203, China

**Keywords:** P-gp-mediated drug–drug interaction, PI3Kα inhibitors, dorzagliatin, LC-MS/MS analysis, PK/PD models

## Abstract

**Objectives:** The anticancer effects of PI3Kα inhibitors (PI3Ki) are constrained by their hyperglycemic side effects, while the efficacy of conventional hypoglycemic agents, such as insulin, metformin, and SGLT-2 inhibitors, in mitigating PI3Ki-induced hyperglycemia remains suboptimal. Dorzagliatin, a novel glucokinase activator, has been approved in China for the management of hyperglycemia, offering a promising alternative. This study aims to investigate the pharmacokinetic properties and potential mechanisms of drug interactions of dorzagliatin in the regulation of PI3K-induced hyperglycemia. **Methods:** Plasma concentrations of WX390, BYL719, and Dorz in mice were measured using high performance liquid chromatography-tandem mass spectrometry (LC-MS/MS) analysis. Pharmacokinetic (PK) parameters and PK/PD models were derived by using Phoenix WinNonlin 8.3.5 software. Blood glucose levels at various time points and tumor volume changes over a four-week period were assessed to explore the interactions when PI3Ki were combined with dorzagliatin. **Results:** The results indicated that, compared to the Dorz group, the combination groups (Dorz + BYL719, Dorz + WX390) exhibited increases in ${AUC}_{0\to t}$ of dorzagliatin by 41.65% and 20.25%, and in C_max_ by 33.48% and 13.32%, respectively. In contrast, co-administration of these PI3Ki with dorzagliatin resulted in minimal increase in their plasma exposure. The combination therapy group (Dorz+BYL719) exhibited superior antitumor efficacy compared to the BYL719 group. **Conclusions**: Our findings indicate that the drug–drug interactions (DDIs) between dorzagliatin and multiple PI3Ki (including WX390 and BYL719) may partially account for the enhanced antitumor efficacy observed in the combination therapy group compared to PI3Ki monotherapy. This interaction may be explained by the inhibition of P-glycoprotein (P-gp) and the pharmacological mechanism of dorzagliatin regarding the activation of insulin regulation.

## 1. Introduction

### 1.1. Primary Adverse Effects of PI3Ki: Hyperglycemia

*PIK3CA* mutations are frequently observed across various malignancies, including breast cancer, endometrial cancer, and ovarian cancer, highlighting the potential of PI3Ki as a promising therapeutic strategy [1,2]. Targeted therapies that inhibit the PI3K pathway have shown substantial antitumor activity in both in vitro and in vivo models of ovarian cancer, underscoring their clinical relevance [3,4]. Clinical trials involving PI3Ki, whether used as monotherapy or in the combination with other targeted agents, have demonstrated significant efficacy in treating late-stage solid tumors harboring *PIK3CA* mutations [5,6,7,8]. Despite their therapeutic potential, hyperglycemia remains one of the most common adverse effects associated with PI3Ki [5,9,10]. In the SOLAR-1 trial, a significant proportion of patients treated with Alpelisib (BYL719) experienced hyperglycemia, with rates of 63.7% for any grade and 36.6% for grades 3–4. The incidence of hyperglycemia contributed to a treatment discontinuation rate of 6.3% [11]. Similarly, WX390, a potent dual inhibitor of PI3K and mTOR [12], shows a comparable incidence of hyperglycemia in its clinical trials [13].

### 1.2. Limitations of Current Hyperglycemia Management Strategies

The hyperglycemia induced by PI3Ki can lead to compensatory increases in insulin secretion, which have been shown to reactivate the PI3K/AKT signaling pathway within tumor cells, potentially inducing resistance to therapy [14]. Therefore, the effective management of hyperglycemia is crucial for maintaining patient weight and quality of life during cancer treatment. Various strategies have been proposed to manage PI3K inhibitor-induced hyperglycemia, including the administration of exogenous insulin. However, this approach may further diminish the antitumor efficacy of PI3Ki and facilitate tumor progression [15]. Other insulin-sensitizing agents exhibit similar limitations.

### 1.3. Dorzagliatin: A Novel Alternative for PI3Ki-Induced Hyperglycemia

In light of these challenges, dorzagliatin, a novel glucose kinase activator (GKA), emerges as a promising alternative. Dorzagliatin has the potential to restore glucose homeostasis and promote glycogen synthesis, thus offering a viable solution for managing hyperglycemia [16,17]. Additionally, it enhances pancreatic β-cell function, leading to the improvement of insulin secretion and rapid regulation of blood glucose levels. This dual mechanism not only stabilizes blood glucose but also minimizes the systemic effects of insulin [18]. Following its approval in China in September 2022, dorzagliatin is now being used both as a monotherapy and in combination with metformin for adults with type 2 diabetes mellitus (T2DM). This positions dorzagliatin as an attractive candidate for the management of PI3K inhibitor-induced hyperglycemia, with the potential to sustain the therapeutic efficacy of PI3Ki without the drawbacks associated with insulin feedback. Our research team has previously demonstrated this perspective in both preclinical and clinical studies investigating the ability of dorzagliatin to mitigate hyperglycemia induced by PI3K inhibitors [19]. However, it is yet to be determined whether this phenomenon is associated with drug–drug interactions. We are endeavoring to approach this issue from a new pharmaceutical perspective.

In this context, the objective of this study is to develop a sensitive LC-MS/MS method for the determination of plasma concentrations of WX390, BYL719, and dorzagliatin. We aim to investigate the pharmacokinetic parameters and pharmacodynamic (PD) effects of dorzagliatin in mouse models, exploring its interactions with PI3Ki. By assessing plasma glucose levels, tumor volume and weight in OVCAR3 xenograft tumor-bearing nude mice, we intend to elucidate the influence of dorzagliatin on the pharmacokinetics of PI3Ki and its potential to enhance their therapeutic benefits. This study represents a pioneering investigation into the pharmacokinetic and drug interactions of WX390, BYL719, and dorzagliatin, with implications for improving treatment outcomes in patients undergoing PI3K inhibitor therapy.

## 2. Results

### 2.1. LC-MS/MS Analysis

**Figure 1:**
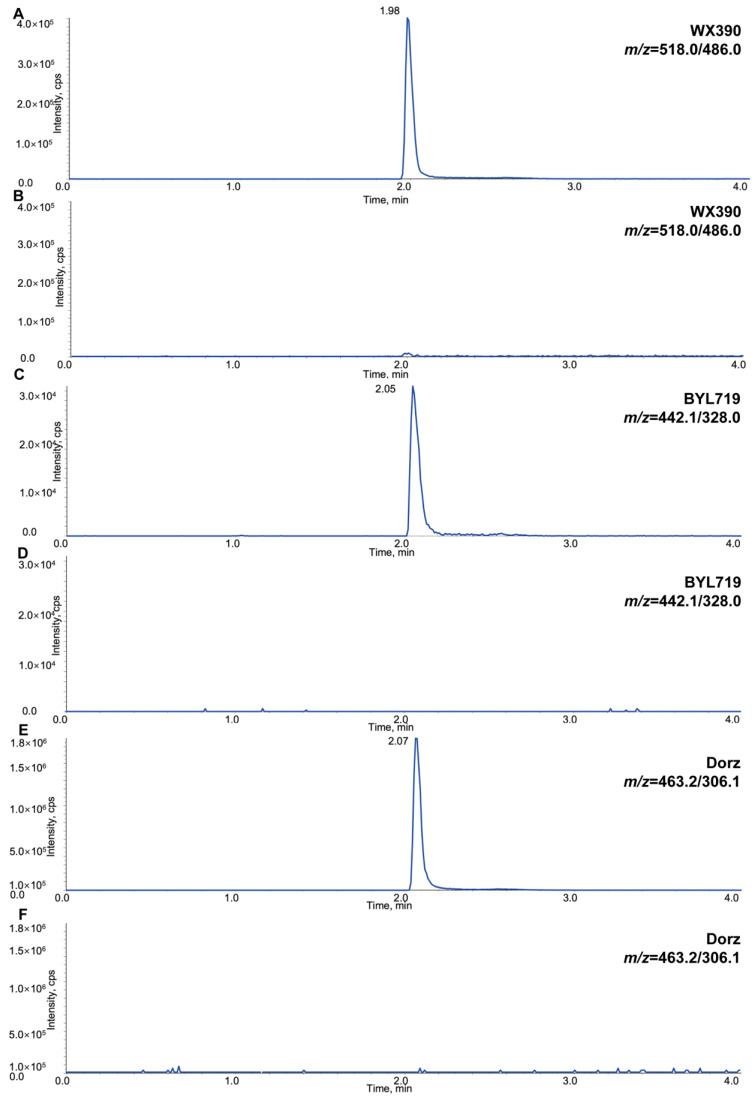

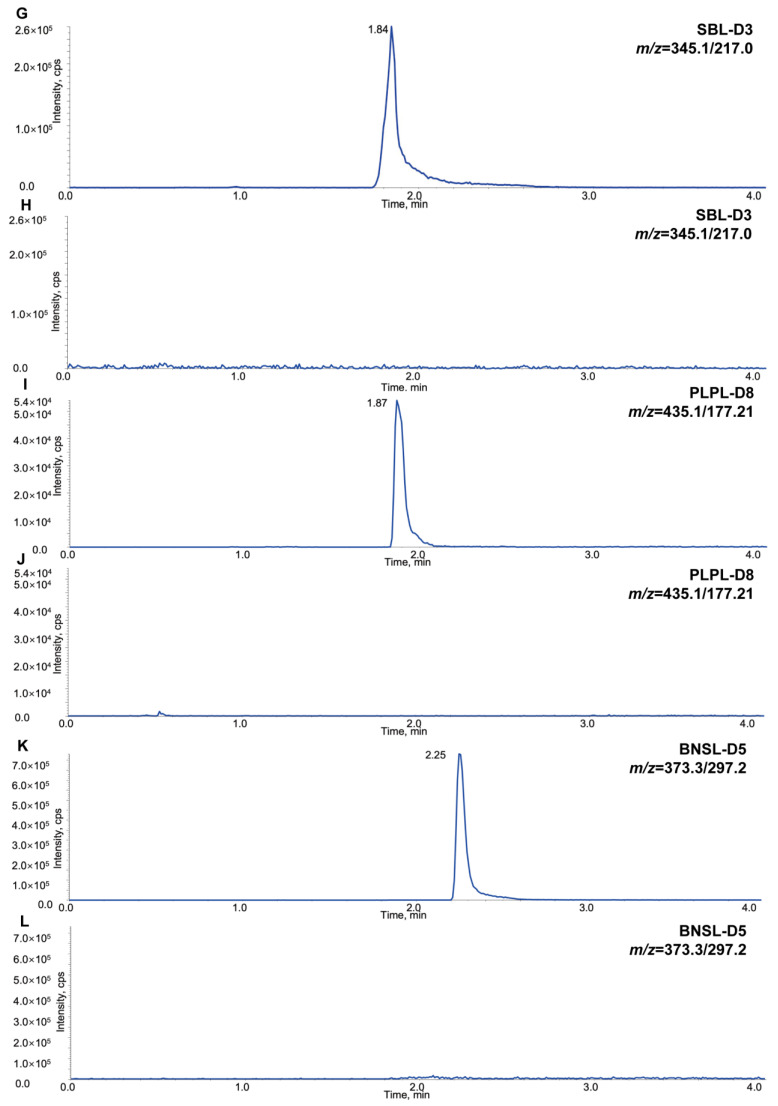
Typical multiple reaction monitoring chromatograms of samples in mouse plasma and Blank mouse plasma. (**A**) WX390 (LLOQ = 0.1 ng/mL), and (**B**) blank control of WX390; (**C**) BYL719 (LLOQ = 1.0 ng/mL), and (**D**) blank control of BYL719; (**E**) dorzagliatin (LLOQ = 2.0 ng/mL), and (**F**) blank control of dorzagliatin; (**G**) Sulpiride-D3 (SBL-D3, 2.0 ng/mL), and (**H**) blank control of SBL-D3; (**I**) Perospirone-D8 (PLPL-D8, 1.0 ng/mL), and (**J**) blank control of PLPL-D8; (**K**) Blonanserin-D5 (BNSL-D5, 3.0 ng/mL), and (**L**) blank control of BNSL-D5.

**Figure 2:**
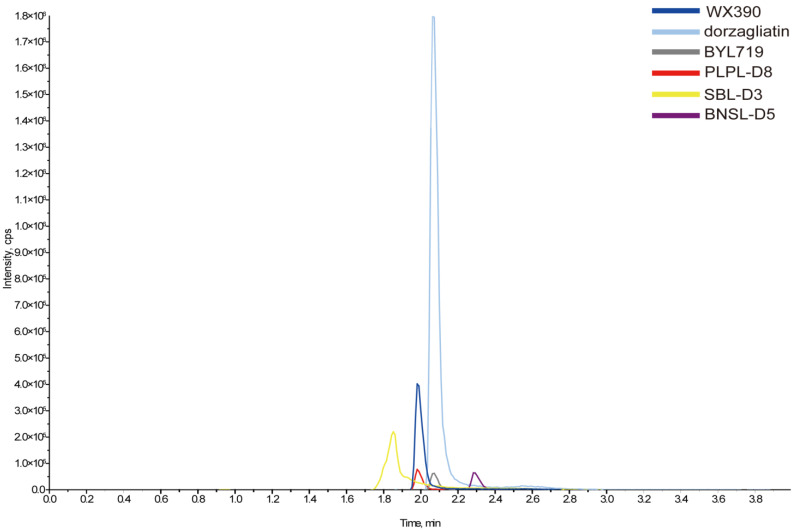
Representative MS/MS chromatogram of six components in mouse plasma.

A novel LC-MS/MS assay was successfully developed and validated for the simultaneous quantification of WX390, BYL719, and dorzagliatin in mouse plasma. This method demonstrated high sensitivity, specificity, and reproducibility. Representative chromatograms of blank mouse plasma, blank plasma spiked with WX390, BYL719, and dorzagliatin, as well as their internal standards (Sulpiride-D3, Blonanserin-D5, and Perospirone-D8), are shown in Figure 1 and Figure 2. These chromatograms illustrate the clear separation and precise detection of the compounds and their respective internal standards. The calibration curves for WX390, BYL719, and dorzagliatin were linear over the concentration ranges of 0.1–200 ng/mL, 1.0–2000 ng/mL, and 2.0–4000 ng/mL, respectively, with correlation coefficients (*r*^2^) consistently greater than 0.99. The lower limits of quantification (LLOQ) were determined to be 0.1 ng/mL for WX390, 1.0 ng/mL for BYL719, and 2.0 ng/mL for dorzagliatin, indicating the assay’s high sensitivity. The intra-batch and inter-batch accuracy and precision of L-QC, M-QC, and H-QC were evaluated, as shown in Table 1 and Table 2. These parameters were found to be in compliance with the EMA Guideline on Bioanalytical Method Validation. Specifically, the intra-assay and inter-assay accuracy (mean % bias) at each QC level did not exceed 15%. Furthermore, the intra-batch and inter-batch precision (%CV) for the low, medium, and high QC levels was also within the acceptable limit of ≤15%. The assay requires only 20 µL of plasma per sample, minimizing the sample volume needed for analysis. Protein precipitation was employed as a simple and effective sample preparation method, yielding consistent and robust extraction recoveries. The recovery and accuracy values for all three compounds were within acceptable ranges as the EMA’s guidelines. Furthermore, the total analytical run time was optimized to minutes, ensuring the rapid and efficient quantification of the compounds. These results highlight the reliability and efficiency of the LC-MS/MS assay for the simultaneous analysis of WX390, BYL719, and dorzagliatin in preclinical pharmacokinetic studies.

### 2.2. Effect of Dorzagliatin on WX390 and BYL719 Pharmacokinetics in Nude Mice

**Figure 3:**
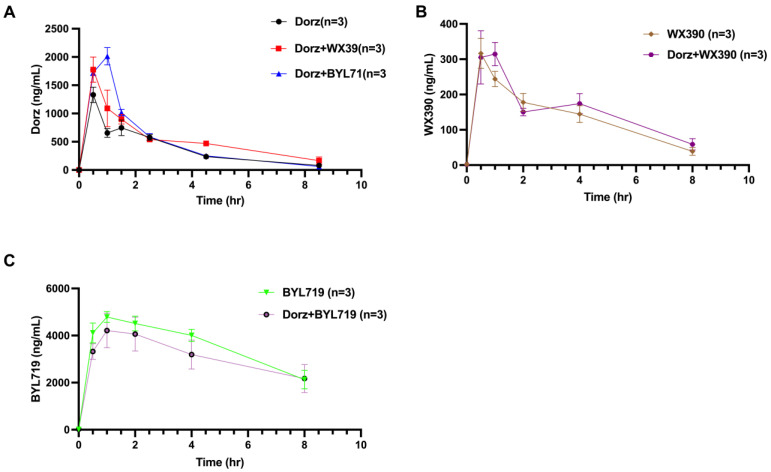
The Concentration-Time curves of WX390, BYL719 and dorzagliatin (**A**) Dorz (10 mg/kg), Dorz (10 mg/kg) + BYL719 (25 mg/kg) and Dorz (10 mg/kg) + WX390 (0.2 mg/kg), (**B**) WX390 (0.2 mg/kg) and Dorz (10 mg/kg) + WX390 (0.2 mg/kg), and (**C**) BYL719 (25 mg/kg) and Dorz (10 mg/kg) + BYL719 (25 mg/kg) in OVCAR3 xenograft tumor-bearing nude mice, administered via oral gavage once daily for four weeks. The curves represent the plasma concentration of three drugs over time.

In the PK interaction study, we administered dorzagliatin (10 mg/kg), BYL719 (25 mg/kg), and WX390 (0.2 mg/kg) to mice, with doses calculated based on established human therapeutic doses and previous animal studies [12,20,21]. The pharmacokinetic parameters of both BYL719 and WX390 remained largely unchanged in the combination treatment groups compared to their respective monotherapy groups. However, a notable finding was that co-administration of PI3Ki with dorzagliatin resulted in significantly elevated plasma concentrations of dorzagliatin compared to dorzagliatin monotherapy (Figure 3).

The combined treatment with PI3Ki led to enhanced systemic exposure of dorzagliatin, as evidenced by increased AUC values compared to the control group (dorzagliatin alone), detailed in Table 3. Specifically, the ${AUC}_{0\to t}$ of dorzagliatin showed significant increases of 20.25% in the Dorz + BYL719 group and 41.65% in the Dorz + WX390 group. Similarly, C_max_ was elevated by 13.32% and 33.48% in these respective combination groups.

### 2.3. Effect of Dorzagliatin on Tumor Levels in OVCAR3 Xenograft Tumor-Bearing Nude Mice

**Figure 4:**
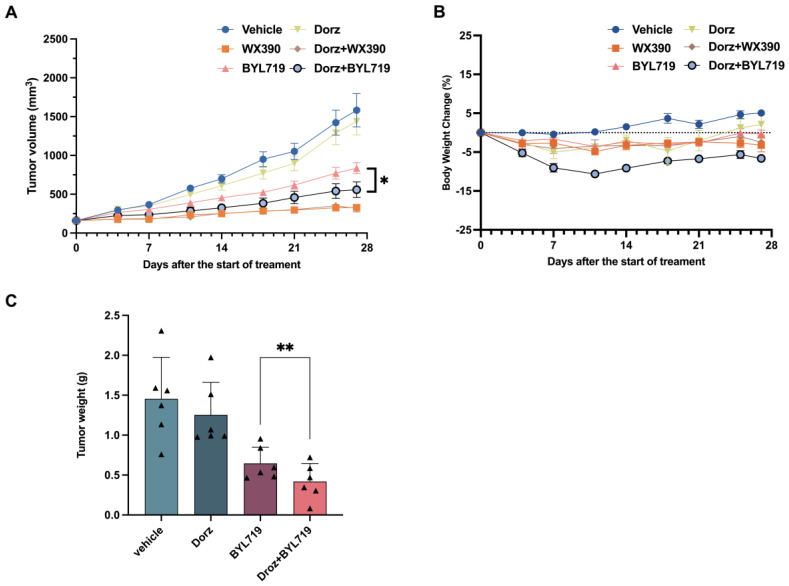
Effect of dorzagliatin on tumor levels in OVCAR3 xenograft tumor-bearing nude mice (**A**) Tumor growth curves of OVCAR3 cell subcutaneous xenograft tumor model in BALB/c mice after administration of the test treatment. (**B**) Relative body weight change (%). Relative body weight change was calculated based on the weight of the animal at the start of dosing. Data points represent intra-group means and error bars represent standard errors (SEM). (**C**) Statistical analysis of tumor weight on day 28. Each triangle represents a parameter value of an individual animal. * *p* < 0.05, ** *p* < 0.01. Data were analyzed by one-way ANOVA.

On day 28 post-administration, the tumor volumes in vehicle control group reached 1582 mm^3^. Compared to the vehicle control group, WX390 group, Dorz + WX390 group, BYL719 group and Dorz + BYL719 group showed reduced volumes of 329 mm^3^ (T/C = 20.81%, TGI = 88.12%, *p* < 0.05), 318 mm^3^ (T/C = 20.10%, TGI 88.90%, *p* < 0.05), 836 mm^3^ (T/C = 52.85%, TGI = 52.46%) and 559 mm^3^ (T/C = 35.36%, TGI = 71.93%, *p* < 0.05), respectively, indicating antitumor effects (Table 4). In WX390 group and Dorz + WX390 group, tumor regression was more pronounced than regression in BYL719 group and Dorz + BYL719 group (Figure 4A). Of note, the combination of Dorz/BYL719 inhibited tumor growth in vivo more potently than BYL719 monotherapy (*p* < 0.05, Figure 4A). Tumor weight results were consistent with volume measurements (*p* < 0.01, Figure 4C). There were no changes in the mean body weights between the experimental groups (Figure 4B). Nutritional support was provided to all experimental groups from day 10 until the end of the study. No morbidity or mortality occurred among animals.

### 2.4. Effect of Dorzagliatin on Blood Glucose Levels in Nude Mice of PI3Ki-Induced Hyperglycemia

**Figure 5:**
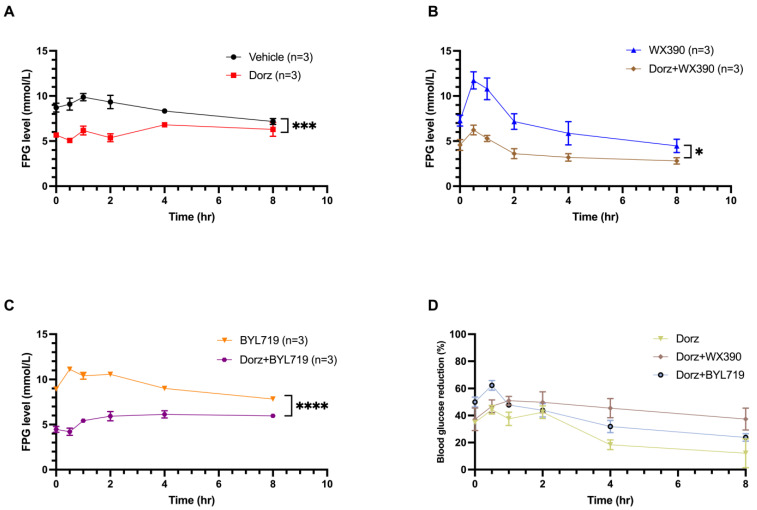

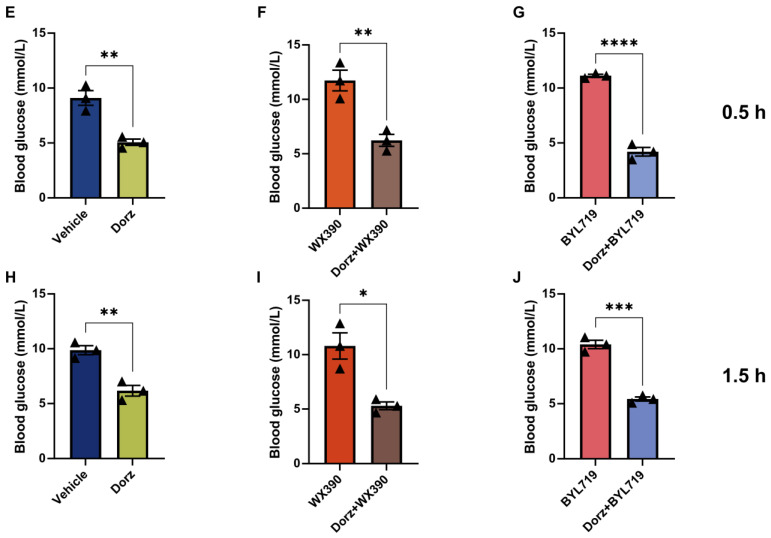
FPG (fasting plasma glucose)-Time curve of (**A**) vehicle vs. Dorz group; (**B**) WX390 vs. Dorz + WX390 group; (**C**) BYL719 vs. Dorz + BYL719 group. (**D**) Blood glucose reduction (∆FPG, the percent reduction in blood glucose levels, %) of Dorz, Dorz + WX390 and Dorz + BYL719 group. Statistical analysis of Blood glucose level at (**E**–**G**) 0.5 and (**H**–**J**) 1.5 h after administration. Each triangle represents a parameter value of an individual animal. * *p* < 0.05, ** *p* < 0.01, *** *p* < 0.001, ***** *p* < 0.0001. Data was analysed by unpaired *t* test.

To test if dorzagliatin can help to manage the blood glucose levels induced by PI3Ki we used a mouse model of PI3K inhibitor-induced hyperglycemia. Blood glucose levels in mice treated with PI3Ki showed significant elevation (*p* < 0.001, Figure 5A) compared to the control. However, plasma glucose levels in the mice treated with the combination (Dorz + WX390 and Dorz + BYL719) showed less elevation (*p* < 0.05, Figure 5B, and *p* < 0.0001, Figure 5C). The mean glucose levels in the Dorz + BYL719 group ranged from 4.20 to 6.13 mmol/L across all time points, while the Dorz + WX390 group showed a range of 2.80 to 6.23 mmol/L. The administration of dorzagliatin showed a substantial reduction in PI3K inhibitors-induced hyperglycemia compared to the control groups at 0.5 and 1.5 h (*p* < 0.01, Figure 5E–G and *p* < 0.05, Figure 5H–J).

### 2.5. PK/PD Research

**Figure 6:**
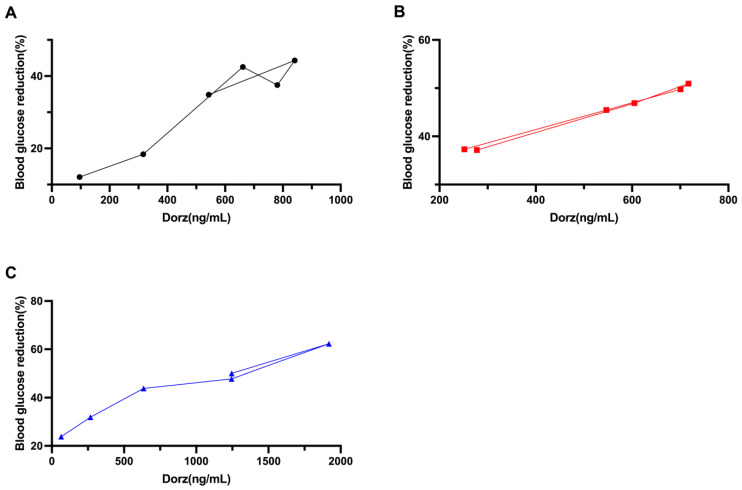
The effect–concentration curves of (**A**) Dorz, (**B**) Dorz + WX390, and (**C**) Dorz + BYL719 group.

The PK/PD simulation was then carried out by employing the final collected data. The effect-concentration curves of Dorz, Dorz + WX390 and Dorz + BYL719 group are shown in Figure 6. A sigmoid Emax model provided the most accurate descriptions of dorzagliatin concentration and ∆FPG, with calculated parameters for dorzagliatin, were determined using this model. Table 5 displays the final quantified PK/PD formulas for each group that incorporate drug levels and ∆FPG.

**Figure 7:**
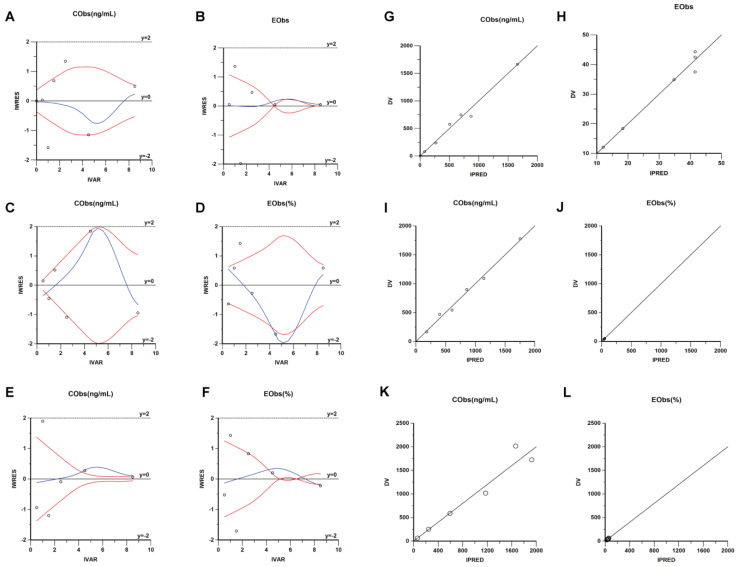
Diagnostic plots for Dorzagliatin PK/PD models. **1. Dorz group**: (**A**) PK time-weighted residuals; (**B**) PD time-weighted residuals; (**G**) observed vs. predicted PK; (**H**) observed vs. predicted PD. **2. DORZ + WX390 group:** (**C**) PK time-weighted residuals; (**D**) PD time-weighted residuals; (**I**) observed vs. predicted PK; (**J**) observed vs. predicted PD. **3. DORZ + BYL719 group**: (**E**) PK time-weighted residuals; (**F**) PD time-weighted residuals; (**K**) observed vs. predicted PK; (**L**) observed vs. predicted PD. The black solid line represents the baseline value of 0, the red solid line indicates the fitted line, the black dashed line serves as an auxiliary line for |IWRES| = 2, and the circles denote the observed values.

Through diagnostic plots and visual predictive checks, the PK and PD models for dorzagliatin, with ∆FPG as the efficacy endpoint, were evaluated. As shown in Figure 7, the PK and PD model demonstrates good accuracy in predicting observed drug concentrations and drug effects. The final three fitted models are stable, with trend lines closely matching standard lines, indicating a strong correlation between IPRED and observed drug effects. In the IWRES vs. Time scatter plot, the points are evenly distributed around the zero line, with most points lying within ±2, further confirming the excellent fit of the PD model to the observed data.

Using ∆FPG as an indicator, the EC50 values for the Dorz group, Dorz + WX390 group, and Dorz + BYL719 group are 740 ng/mL, 490 ng/mL, and 660 ng/mL, respectively, with Hill coefficients (γ) of 0.80, 1.35, and 0.95, respectively. Through the PK/PD model, we observed a synergistic advantage of WX390 when combined with Dorz. The EC50 of the Dorz + WX390 group decreased by 34% compared to the Dorz monotherapy group, supporting its role as a potentiator for dorzargliatin. In contrast, the EC50 reduction in the Dorz + BYL719 group was less pronounced (11%). Additionally, the Hill coefficient indicated that the response curve for the Dorz + WX390 group was steeper (γ = 1.35), suggesting enhanced synergism. This implies that WX390 may facilitate the binding of dorzagliatin to its receptor or amplify downstream signaling. The Dorz + BYL719 group also exhibited increased synergism, but to a lesser extent (γ = 0.95), indicating a minimal impact of BYL719 on the binding kinetics of dorzagliatin.

## 3. Discussion

Both WX390 and BYL719 are inhibitors of PI3K, targeting the PI3K-mTOR signaling pathway, which plays a critical role in tumor growth and survival. These inhibitors exhibit significant antineoplastic properties and are being actively explored in cancer therapy, particularly in tumors with *PIK3CA* mutations. However, a notable and clinically relevant side effect of PI3Ki is the occurrence of hyperglycemia, which arises due to the disruption of insulin signaling pathways. To address this challenge, we explored the use of dorzagliatin, a novel glucose-lowering agent, as a potential therapeutic strategy to mitigate PI3Ki-induced hyperglycemia.

### 3.1. The Advantages of Determining Dorzagliatin, WX390 and BYL719 by LC-MS/MS Method

LC-MS/MS is widely recognized as a highly sensitive and reliable analytical technique for quantifying drugs at low concentrations in plasma. Several LC-MS/MS methods have been reported for the determination of BYL719 and dorzagliatin concentrations in plasma [22,23]. However, these methods are often limited by operational complexity and lengthy processing, which can extend up to 5 h [23]. To overcome these limitations, we developed a rapid and efficient LC-MS/MS method for the simultaneous quantification of WX390, BYL719, and dorzagliatin. Notably, this study is the first to report the measurement of WX390 concentrations in mouse plasma, marking a significant advancement in bioanalytical methodologies.

Compared to previously published methods for BYL719 and dorzagliatin [23,24], which typically involve protein precipitation using acetonitrile or tert-butyl methyl ether followed by nitrogen drying at 40 °C [25], our method employs a simple 0.1 mol/L zinc sulfate aqueous solution for protein precipitation. This novel approach demonstrates multiple advantages in analytical chemistry. Firstly, cost-effectiveness is achieved through the substitution of conventional organic solvents with zinc sulfate. Secondly, environmental sustainability is enhanced by reducing volatile organic compound emissions, thereby conforming to green chemistry principles. Thirdly, operational safety is significantly improved through the elimination of nitrogen drying procedures and hazardous solvent handling. Most notably, workflow efficiency is optimized via streamlined sample preparation protocols, enabling high-throughput analysis capabilities.

These improvements address key considerations in the development of sustainable and responsible bioanalytical techniques, making this method a valuable tool for both preclinical and clinical research. The dosing regimen for these compounds was carefully designed based on their clinical use in humans. Using allometric scaling, we calculated the human equivalent dose for mice, normalizing the conversion to body surface area in accordance with established methodologies [26]. This approach ensures the translational relevance of the findings, bridging the gap between preclinical and clinical research.

### 3.2. An Overview of the Pharmacokinetic Profiles of the Three Investigated Pharmaceuticals

Existing evidence indicates that the pharmacokinetic profile of dorzagliatin is consistent between healthy individuals and patients with T2DM. After oral administration, dorzagliatin is rapidly absorbed, with a median Tₘₐₓ of 1.31 h, Cₘₐₓ of 1090 ng/mL, and AUC_tau_ of 5570 h·ng/mL [27]. In T2DM patients, twice-daily oral administration achieves steady-state plasma pharmacokinetics by day 4, with an average accumulation ratio of 1.0 to 1.8 compared to a single dose [28]. Following a single 50 mg oral dose in healthy subjects, dorzagliatin displays a mean apparent volume of distribution of 115 L [25]. Metabolism primarily occurs in the liver via cytochrome P450 enzyme 3A4 (CYP3A4) [29].

In contrast, the pharmacokinetic parameters of WX390 remain undisclosed, highlighting a gap in the available data. However, insights can be drawn from other PI3Ki, such as BYL719, which was approved by the U.S. FDA in May 2019. Under fed conditions, the exposure of BYL719 increases proportionally with doses ranging from 30 to 450 mg. At a clinically relevant dose of 300 mg/day, BYL719 achieves a Cₘₐₓ of 2480 ng/mL, an ${AUC}_{0\to 24h}$ of 33,224 ng·h/mL, and reaches peak plasma levels within 2–4 h [30,31]. Food intake does not significantly affect the pharmacokinetic profile of BYL719. The apparent volume of distribution of BYL719 is 114 L, with a high plasma protein binding rate of 89%, a half-life of 8–9 h, and a clearance rate of 9.2 L/h [31].

In vitro studies reveal that BYL719 is primarily metabolized through chemical degradation and enzymatic hydrolysis, with the major metabolite identified as BZG791. CYP3A4-mediated metabolism contributes to a lesser extent. These pharmacokinetic characteristics of BYL719 provide a useful reference for understanding the behavior of similar PI3K inhibitors such as WX390, particularly in the absence of publicly available data on its pharmacokinetics.

### 3.3. Mechanisms for Drug Drug Interaction Between Dorzagliatin and PI3Ki

#### 3.3.1. CYP3A4 Does Not Contribute to the Enhanced Exposure of Dorzagliatin

In the combination treatment groups (Dorz + BYL719 and Dorz + WX390), the ${AUC}_{0\to t}$ of dorzagliatin was significantly higher compared to the group receiving dorzagliatin alone, with increases of 41.65% and 20.25%, respectively. According to the prescribing information for dorzagliatin, potent inhibitors of CYP3A4 can significantly enhance its systemic exposure. Co-administration with moderate CYP3A4 inhibitors such as verapamil, fluconazole, and erythromycin has been shown to result in a 2.4-fold increase in ${AUC}_{0\to t}$ and a 1.2-fold increase in Cmax of dorzagliatin [32]. However, in vitro studies have demonstrated that dorzagliatin does not inhibit CYP1A2, 2A6, 2C9, 2C19, 2D6, 2E1, or CYP3A4/5, nor does it induce these enzymes to a clinically significant extent, except for CYP1A2, 2B6, 2C9, and 3A4 [32]. Furthermore, dorzagliatin is known to be a substrate of P-gp [27], which may influence its pharmacokinetics.

BYL719 has also been reported to exhibit altered exposure in the presence of CYP3A4 inhibitors, warranting caution during co-administration [33]. However, studies evaluating the pharmacokinetic interactions of repeated doses of 300 mg BYL719 with single doses of sensitive substrates for CYP3A4 (midazolam), CYP2C8 (repaglinide), CYP2C9 (warfarin), CYP2C19 (omeprazole), and CYP2B6 (bupropion) in a cocktail format did not reveal clinically significant interactions. Additionally, no significant differences in the pharmacokinetics of everolimus, a substrate of both CYP3A4 and P-gp, were observed when co-administered with BYL719 [31]. These findings indicate that BYL719 is not a CYP3A4 inhibitor and suggest that the observed increase in dorzagliatin exposure in the combination groups is unlikely to be attributable to CYP3A4 inhibition.

The mechanism underlying the increased exposure of dorzagliatin when co-administered with BYL719 or WX390 remains unclear. It is possible that other factors, such as modulation of P-gp or altered absorption dynamics, may contribute to this effect. Further studies are warranted to investigate the potential pharmacokinetic interactions and mechanisms involved in these combinations to ensure optimal dosing strategies and minimize the risk of adverse effects.

#### 3.3.2. The P-gp Is Likely the Principal Factor Contributing to the Elevation of AUC for Dorzagliatin

The observed increase in dorzagliatin plasma concentration upon co-administration with BYL719 is likely attributable to dorzagliatin being a substrate of P-glycoprotein, while BYL719 acts as a P-gp inhibitor [27,31,32]. P-gp, a member of the ATP-binding cassette (ABC) transporter family, is a 170 kDa ATP-dependent efflux protein of MDR1 encoded by the ABCB1 gene [34]. It is widely expressed in various tissues, including the intestines, liver, brain, and kidneys, where it plays an essential role in limiting drug absorption and distribution due to its broad substrate specificity. Of particular importance is its localization at the apical membrane of intestinal epithelial cells, where it restricts the intestinal absorption of substrate drugs [35,36]. Given the critical role of P-gp in drug pharmacokinetics, regulatory agencies, including the FDA and EMA, recommend early investigation of P-gp substrate or inhibitor interactions during preclinical drug development [37,38]. Furthermore, P-gp is known to significantly reduce the oral bioavailability of certain molecules beyond Rule of 5 (bRo5), highlighting its impact on DDI potential [39].

Accurately predicting P-gp-mediated intestinal absorption and DDIs in humans is a growing priority in drug discovery and development. However, substantial interspecies differences in the types, expression, functional activity, and tissue distribution of drug-metabolizing enzymes (DMEs) and transporters limit the predictive value of conventional animal models [40]. Although novel models, such as knockout (KO) or transgenic rodents with humanized DMEs and/or transporters and chimeric animals with humanized livers, have been developed to better mimic human drug metabolism, these models are not without limitations. In our study, native animal models were used, which, while providing initial insights into the observed increase in dorzagliatin plasma concentration during combination therapy with BYL719, offer limited predictive value for human P-gp function. To address this limitation, our research team plans to employ human-derived models, including Caco-2 permeability assays and P-gp vesicular transport studies, to further elucidate the mechanistic basis of drug–drug interactions.

The clinical implications of this interaction warrant careful consideration, particularly due to the increased risk of hypoglycemia associated with higher dorzagliatin plasma concentrations. However, dorzagliatin’s mechanism of action, which enhances glucose-stimulated insulin secretion and GLP-1 release while improving β-cell function and reducing insulin resistance in type 2 diabetes patients, may mitigate the risk of severe hypoglycemia. A randomized, double-blind, placebo-controlled phase 3 trial of dorzagliatin in drug-naïve patients with type 2 diabetes demonstrated its safety profile: During the 24-week double-blind treatment period, only 1 of 310 patients (0.3%) in the dorzagliatin group reported clinically significant hypoglycemic events (blood glucose < 54 mg/dL). Over the 52-week observation period, the exposure-adjusted event rate for clinically significant hypoglycemia was 0.006 events per patient-year [17]. The results of this study also show that the types and incidence rates of adverse events and serious adverse events were similar to those during the double-blind treatment period. Nevertheless, the known adverse drug reactions (ADRs) with an incidence rate of greater than or equal to 0.5% include: elevated alanine aminotransferase, elevated liver enzymes, elevated transaminases, elevated gamma-glutamyltransferase, hypertriglyceridemia, and dyslipidemia. These adverse reactions are noteworthy when considering combination therapy.

Dorzagliatin’s ability to improve glucose homeostasis positions it as a promising candidate for combination therapy with PI3Ki like BYL719 and WX390, potentially extending the therapeutic window of PI3Ki by alleviating its hyperglycemia-associated adverse effects. Future studies should focus on further characterizing the pharmacokinetic and pharmacodynamic interactions between these agents to optimize their combined clinical use and ensure patient safety.

#### 3.3.3. Dorzagliatin Enhances the Anti-Tumor Efficacy of PI3Ki

Our results clearly showed that dorzagliatin pretreatment significantly reduced the PI3Ki-induced increase in blood glucose levels in mice, suggesting its potential to mitigate hyperglycemia. Additionally, combining dorzagliatin with PI3Ki enhanced antitumor efficacy without increasing the plasma exposure of the inhibitors. We propose that the unique mechanism of dorzagliatin significantly enhances the efficacy of PI3Ki. Our previous studies suggest that the specific activation of hepatic glucokinase and the regulation of insulin levels may underlie its beneficial effects [19]. The enhanced anticancer efficacy observed with the combination therapy is likely mediated through the intricate interplay of multiple signaling pathways, with particular emphasis on the AKT/INSR/mTOR axis [41]. Dorzagliatin’s ability to enhance insulin regulation is instrumental in preventing the hyperactivation of this pathway, thereby facilitating more effective suppression of tumor growth by PI3Ki. Moreover, by optimizing the metabolic state, dorzagliatin may alleviate metabolic stress on tumor cells, thereby enhancing their susceptibility to the cytotoxic effects of PI3K inhibition.

The insights garnered from this study underscore the significant potential of dorzagliatin as an adjunctive therapy for the management of PI3K inhibitor-induced hyperglycemia in cancer patients. By concurrently improving glycemic control and augmenting the antitumor efficacy of PI3Ki, dorzagliatin emerges as a promising candidate for integration into combination therapy regimens. This strategic approach holds the potential to expand the therapeutic window of PI3Ki, enabling the administration of higher doses or extended treatment durations while mitigating the risk of severe hyperglycemia [5]. Such advancements could pave the way for more effective and safer cancer treatments, ultimately enhancing patient outcomes.

## 4. Materials and Methods

### 4.1. Test Compounds and Materials

BYL719 (MW 441.47 g/mol, with purity of ~99.96%) and dorzagliatin (MW 462.93 g/mol, with purity of ~99.90%) were purchased from Selleck Chemicals (Shanghai, China). WX390 (MW 517.58 g/mol, with purity of ~99.90%) was obtained from Jiatan Pharmatech (Shanghai, China). HPLC grade reagents and all other chemicals were obtained from Sigma–Aldrich (Shanghai, China).

### 4.2. Measurement of WX390, BYL719, and Dorzagliatin Levels in OVCAR3 Xenograft Tumor-Bearing Nude Mice

#### 4.2.1. Animals

This study, approved by Fudan University Ethics Committee (protocol code: 201904008Z) on 26 April 2019, was conducted in compliance with AAALAC’s International Commission guidelines for laboratory animal evaluation and accreditation. A total of 36 female BALB/c nude mice (6–8 weeks old, weighing 18–20 g) were purchased from Charles River Laboratories (Pinghu, China). The OVCAR3 ovarian cancer cell line (ATCC^®^ HTB-161™) was used to establish human xenografts. OVCAR3 cells (10 × 10^6^) suspended in 0.2 mL of Matrigel were subcutaneously inoculated into the dorsal region of each mouse. Tumors were allowed to grow until reaching a volume of ~160 mm^3^, typically around 30 days post-inoculation.

#### 4.2.2. Oral Gavage Formulation

WX390 was prepared in Solvent-1, comprising dimethyl sulfoxide (DMSO): methylcellulose: water at a ratio of 1:1:98 (*v/v/v*). BYL719 and dorzagliatin were formulated in Solvent-2, consisting of Tween 80: water with 0.5% methylcellulose (MC) solution at a ratio of 0.2:99.8 (*v/v*). The treatment control was a combination of Solvent-1 and Solvent-2. The test compounds were administered orally as follows: WX390 at 0.2 mg/kg, BYL719 at 25 mg/kg, and dorzagliatin at 10 mg/kg, dissolved in their respective formulations. Due to ethical considerations regarding gavage volume the administration volume across all groups was standardized to 10 μL/g of body weight.

#### 4.2.3. In Vivo Tumor Growth Experiments

The experiment involved six treatment groups, each comprising six mice. The groups included: (i) vehicle control, (ii) WX390 (0.2 mg/kg), (iii) BYL719 (25 mg/kg), (iv) dorzagliatin (10 mg/kg), (v) WX390 (0.2 mg/kg) + dorzagliatin (10 mg/kg), and (vi) BYL719 (25 mg/kg) + dorzagliatin (10 mg/kg). All treatments were administered via oral gavage once daily for four weeks. In the combination group of PI3Ki and dorzagliatin, dorzagliatin was administered 30 min prior to PI3Ki.

Following the 4-week treatment period, all experimental groups were fasted 6 h before the last administration until the end of the experiment. Water was not restricted during the fasting period. Blood samples were collected at 0, 0.5, 1, 2, 4, and 8 h after administration. Each mouse had 3 blood collection points, with 50 μL of plasma collected at each point. Mice in each group were allocated to two cohorts: The first cohort underwent blood collection at 0, 1, and 2 h post-administration, while the second cohort was sampled at 0.5, 4, and 8 h post-administration. Terminal blood collection at the 2-h time point for the first cohort was performed via cardiac puncture following euthanasia, during which tumor tissues were concurrently harvested from this cohort. Blood samples were processed through centrifugation (4 °C, 1500× *g* for 10 min) for plasma separation. All plasma and tumor samples were immediately snap-frozen in liquid nitrogen and stored at −80 °C for subsequent analysis.

### 4.3. Quantification of WX390, BYL719, and Dorzagliatin

#### 4.3.1. Sample Preparation

The extraction of WX390, BYL719, and dorzagliatin from plasma samples was performed using protein precipitation. Internal standards (IS) included SBL-D3, BNSL-D5, and PLPL-D8 for WX390, BYL719, and dorzagliatin, respectively. Plasma samples (20 μL) were mixed with 20 μL of zinc sulfate aqueous solution (0.1 mol/L) and 200 µL of a 50% methanol solution containing three internal standards followed by vortexing for 10 min. The samples were centrifuged at 4 °C (15,000× *g* for 5 min). The supernatant (180 μL) was transferred to a clean Eppendorf tube, and a 10 μL aliquot was injected into the LC-MS/MS system.

#### 4.3.2. Determination of WX390, BYL719, and Dorzagliatin by LC-MS/MS

Chromatographic separations were performed using an AB SCIEX Jasper HPLC system coupled with an AB SCIEX Triple Quad 4500MD mass spectrometer (AB SCIEX, USA). A Phenomenex Kinetex C18 column (100 × 3 mm, 2.6 μm) was used at 40 °C. The flow rate was maintained at 0.4 mL/min with a total run time of 4 min. The mobile phase consisted of:

- Solvent A: Water with 5 mM ammonium acetate and 0.1% formic acid.
- Solvent B: Acetonitrile with 5 mM ammonium acetate.
- The gradient conditions were as follows:
- 0–0.3 min: 5–70% Solvent B.
- 0.3–1.0 min: 70% Solvent B.
- 1.0–1.8 min: 70–98% Solvent B.
- 1.8–2.5 min: 98% Solvent B.
- 2.5–2.51 min: 98–100% Solvent B.
- 2.51–3.0 min: 100% Solvent B.
- 3.0–3.1 min: 100–5% Solvent B.
- 3.11–4.0 min:5% Solvent B.

Mass spectrometry parameters were as follows: curtain gas at 30 psi, collision gas at 8, ion spray voltage at 5500 V, source temperature at 550 °C, and ion source gas 1 and 2 at 30 °C. Chromatographic data processing was conducted using Analyst 1.6.3 software, and quantification was performed using MultiQuant™ MD 3.0.2 software.

### 4.4. Tumor Measurement

Tumor dimensions were measured biweekly using a vernier caliper. Tumor volume was calculated using the formula:

(1) $$V=0.5a\times b^{2}$$

where:

- *a* represents the longest tumor diameter.
- *b* represents the shortest diameter.

The antitumor efficacy of each group was evaluated using TGI or T/C. TGI (%) reflects the rate of tumor growth inhibition and is calculated as follows:

(2) $$TGI\left(\%\right)=1-\frac{\left(V_{end}-V_{0}\right)exp}{\left(V_{end}-V_{0}\right)control}\times 100\%$$

where:

- *V_end_* represents mean tumor volume at the end of treatment in the group.
- *V*_0_ represents mean tumor volume at the start of treatment in the group.
- *exp* represents the treated groups.
- *control* represents the vehicle control group.

T/C (%) is calculated using the formula:

(3) $$\frac{T}{C}\left(\%\right)=\frac{T_{RTV}}{C_{RTV}}\times 100\%$$

where:

- *T_RTV_* is the Relative Tumor Volume of the treatment group.
- *C_RTV_* is the Relative Tumor Volume of the negative control group.

The Relative Tumor Volume (RTV) is calculated based on tumor measurement results using the formula:

(4) $$RTV=\frac{V_{t}}{V_{0}}$$

where:

- *V*_0_ is the mean tumor volume at the time of grouping (day 0).
- *V_t_* is the mean tumor volume at a given measurement time.

*T_RTV_* and *C_RTV_* are based on data from the same day.

At the conclusion of the experiment, tumor weights were measured, and the T/C was calculated. Tweight and Cweight represent the tumor weights of the treatment group and the vehicle control group, respectively.

### 4.5. Pharmacodynamic Study Design of Dorzagliatin in Nude Mice

For the pharmacodynamic study, 18 female BALB/c nude mice (6–8 weeks old) were divided into six groups (*n* = 3). Food was systematically removed 6 h prior to the final drug administration, initiating a fasting period with ad libitum water access maintained across all experimental groups until study termination. The grouping, administration methods, and dosages were consistent with those described in Section 4.2. On Day 28, Blood samples (10 μL) were collected from the tail vein at baseline (0 h) and specified intervals (1, 2, 4, 6, and 8 h), with blood glucose concentrations measured using a OneTouch Ultra glucometer. At the end of the experiment, ~100 μL of blood was collected from each mouse into EDTA-coated tubes (Sarstedt, #16.444). The blood samples were centrifuged at 4 °C (10,000× *g* for 10 min), and the plasma was stored at −20 °C.

### 4.6. Model Development

#### 4.6.1. PK Analysis

Plasma concentrations of WX390, BYL719, and dorzagliatin were quantified, and standard curves were generated for each batch of samples to ensure accurate calibration. Dosing information, measured plasma concentrations, and corresponding post-administration time points were input into the Noncompartmental Analysis (NCA) module of Phoenix (Version 8.3.5, Certara, Saint Louis, MO, USA) to estimate pharmacokinetic parameters. Pharmacokinetic variables, such as Cₘₐₓ, Tₘₐₓ, and AUC, were derived directly from the concentration-time data.

To further characterize the pharmacokinetics of the compounds, a two-compartment model with first-order absorption was developed using the Phoenix Model module in Phoenix software. The AIC method was applied to identify the most suitable pharmacokinetic model. This model facilitated the comprehensive evaluation of the pharmacokinetic behavior of the drugs under investigation.

#### 4.6.2. PK/PD Analysis

To evaluate the effect of the test compounds on blood glucose levels, 10 μL of blood was collected from the tail veins of mice at baseline (time 0) and at predetermined time points (0.5, 1, 2, 4, 6, and 8 h) post-administration. Blood glucose levels were measured using a OneTouch Ultra glucometer. Changes in FPG levels over time were monitored and analyzed. ∆FPG was calculated using Equation (5):

(5) $$∆FPG=\frac{{FPG}_{control}-{FPG}_{exp}}{{FPG}_{control}}\times 100\%$$

where:

- *FPG_exp_* represents the FPG levels in the dorzagliatin group, dorzagliatin + WX390 group, or the dorzagliatin + BYL719 group.
- *FPGcontrol* represents the FPG levels in the vehicle control group, WX390 group, or BYL719 group.

A preliminary PK/PD interaction model was developed using plasma concentrations and FPG levels in tumor-bearing mice. The relationship between plasma drug concentrations and FPG reduction was described using the Sigmoid Eₘₐₓ model (Equation (6)):

(6) $$E={E_{0}+E}_{max}\times \frac{{Ce}^{\gamma }}{{EC}_{50}^{\gamma }+{Ce}^{\gamma }}$$

where:

- *E* represents the mean percent blood glucose reduction.
- *Cₑ* indicates the plasma concentration of the drug that elicits a pharmacodynamic response.
- *E*_0_ is the baseline drug effect.
- *Eₘₐₓ* represents the maximum potential drug effect.
- *EC*_50_ is the plasma concentration at which 50% of the maximum drug effect is observed.
- *γ* denotes the Hill coefficient, which reflects the steepness of the concentration-effect curve.

This PK/PD model was constructed to quantitatively describe the interaction between the PI3Ki and dorzagliatin in tumor-bearing mice.

### 4.7. Data Analysis

Comparative analyses between treatment groups were conducted using appropriate statistical tests. Differences in mean values between two groups were assessed using the Student’s *t*-test for parametric data or the Mann–Whitney U test for non-parametric data. All statistical analyses were two-tailed, and a *p*-value of less than 0.05 was considered statistically significant.

## 5. Conclusions

This study represents the pioneering investigation into the pharmacokinetic interplay between PI3Ki and dorzagliatin in a tumor-bearing murine model, revealing a notable increase in dorzagliatin’s systemic exposure. Our findings elucidate a potential mechanism wherein PI3Ki, by inhibiting P-gp, enhances the plasma concentration of dorzagliatin, a known P-gp substrate. The combination therapy of dorzagliatin and PI3Ki not only mitigated the hyperglycemic adverse effects typically associated with PI3Ki but also significantly augmented antitumor efficacy, as evidenced by reduced tumor growth compared to PI3Ki monotherapy. These seminal observations suggest a dual benefit of improved metabolic regulation and enhanced anticancer activity, positioning dorzagliatin as a strategic adjunct to PI3Ki therapy. The findings of this study are derived from preclinical research. Further clinical studies and in vitro model investigations are warranted to validate these results and fully explore the therapeutic potential of this novel combination in clinical oncology, with the aim of optimizing treatment outcomes and patient care.

## 6. Patents

Guanqin Jin, Yu Kang. “A composition of a glucokinase activator and a PI3K inhibitor.” CN2025104313785, 8 April 2025.

## Figures and Tables

**Table 1:** Intra-assay’s accuracy and precision.

Parameter	WX390				BYL719				Dorzagliatin			
Quality control sample (target concentration)	LLOQ 0.1 ng/mL	L-QC 1 ng/mL	M-QC 40 ng/mL	H-QC 200 ng/mL	LLOQ 1 ng/mL	L-QC 10 ng/mL	M-QC 400 ng/mL	H-QC 2000 ng/mL	LLOQ 2 ng/mL	L-QC 20 ng/mL	M-QC 800 ng/mL	H-QC 4000 ng/mL
Number of analyzed samples	10	10	10	10	10	10	10	10	10	10	10	10
Concentration found ng/mL (median, range)	0.10 ± 0.01	1.02 ± 0.03	39.56 ± 1.54	201.78 ± 2.86	1.04 ± 0.12	10.15 ± 0.26	408.42 ± 16.34	2014.14 ± 16.28	1.92 ± 0.265	20.21 ± 0.69	812.84 ± 21.56	4052.16 ± 32.92
Intra-assay %bia	−3.9	1.8	−1.1	0.9	4.1	1.5	2.1	0.7	−3.8	2.1	1.6	1.3
Intra-assay %CV	9.8	3.1	3.9	1.4	11.6	2.6	3.9	0.8	13.9	3.5	2.6	1.7

**Table 2:** Inter-assay’s accuracy and precision.

Parameter	WX390				BYL719				Dorzagliatin			
Quality control sample (target concentration)	LLOQ 0.1 ng/mL	L-QC 1 ng/mL	M-QC 40 ng/mL	H-QC 200 ng/mL	LLOQ 1 ng/mL	L-QC 10 ng/mL	M-QC 400 ng/mL	H-QC 2000 ng/mL	LLOQ 2 ng/mL	L-QC 20 ng/mL	M-QC 800 ng/mL	H-QC 4000 ng/mL
Number of analyzed samples	10	10	10	10	10	10	10	10	10	10	10	10
Concentration found ng/mL (median, range)	0.10 ± 0.01	1.02 ± 0.08	39.58 ± 1.53	198.64 ± 5.42	1.02 ± 0.07	10.18 ± 0.35	403.72 ± 15.48	1992.36 ± 40.88	1.98 ± 0.15	20.32 ± 0.90	808.36 ± 25.22	3984.12 ± 82.86
Inter-assay %bia	−2.1	1.8	−2.0	−0.7	2.0	1.8	0.9	−0.4	−1.5	1.6	1.0	−0.4
Inter-assay %CV	8.7	6.2	4.2	2.1	5.4	2.8	3.1	1.7	6.8	3.5	2.6	1.9

**Table 3:** Main pharmacokinetic parameters of the WX390, BYL719 and dorzagliatin of 5 groups (*n* = 3).

Group	Ingredient	t_1/2_ (h)	T_max_ (h)	C_max_ (ng/mL)	${AUC}_{0\to t}$ (ng × h/mL)
Dorz	Droz	2.15	0.50	1331.00	3287.90
WX390	WX390	2.70	0.50	316.53	1119.16
Dorz + WX390	WX390	3.81	1.00	314.40	1177.53
	Dorz	2.93	0.50	1776.67	4657.23
BYL719	BYL719	5.33	1.00	4786.67	29,035.20
Dorz + BYL719	BYL719	6.72	1.00	4212.00	24,839.06
	Dorz	1.86	1.00	2013.33	3953.58

**Table 4:** Antitumor efficacy evaluation of each group on xenograft tumor model (calculated based on tumor volume on the Day 28 after administration) (*n* = 6).

Group	Tumor Volume (mm^3^) (Day 28)	T/C (%)	TGI (%)	*p* Volue
Vehicle	1582 ± 215	/	/	/
Dorz	1437 ± 172	90.81	10.19	0.994
WX390	329 ± 24	20.81	88.12	0.013
Dorz + WX390	318 ± 48	20.10	88.90	0.012
BYL719	836 ± 71	52.85	52.46	0.104
Dorz + BYL719	559 ± 101	35.36	71.93	0.026

**T/C** means the Relative Tumor Growth Rate. **TGI** means the Tumor Growth Inhibition. *p*-values were calculated based on tumor volume. Data were analyzed by one-way ANOVA.

**Table 5:** PK/PD model equation for dorzagliatin of three groups.

Ingredient	Group	Model	AIC	PK/PD Equation
Dorz	Dorz	Two-Compartment Mode	51.20	E = 10 + 50 × C^1.10^/(740^0.80^ + C^0.80^)
	Dorz + WX390	Two-Compartment Mode	114.92	E = 12 + 56 × C^1.35^/(490^1.35^ + C^1.35^)
	Dorz + BYL719	Two-Compartment Mode	121.36	E = 18 + 60 × C^0.95^/(660^0.95^ + C^0.95^)

## Data Availability

The data presented in this study are available on request from the corresponding author. The data are not publicly available due to intellectual property protection.

## Abbreviations

Pi3Ki	PI3Kα inhibitors
PK-PD	pharmacokinetic-pharmacodynamic
Dorz	Dorzagliatin
P-gp	P-glycoprotein
BYL719	alpelisib
T2DM	type 2 diabetes mellitus
LC-MS/MS	high performance liquid chromatography-tandem mass spectrometry
MC	methylcellulose
NCA	Noncompartmental Analysis
Cₘₐₓ	maximum plasma concentration
Tₘₐₓ	time to reach maximum concentration
AUC	area under the plasma concentration-time curve
AIC	Akaike Information Criterion
ADRs	adverse drug reactions
FPG	fasting plasma glucose
∆FPG	the percent reduction in blood glucose levels
LLOQ	lower limits of quantification
DDI	drug–drug interaction
bRo5	beyond Rule of 5
DMEs	drug-metabolizing enzymes
KO	knockout
